# Antidepressants use and risk of cataract development: a systematic review and meta-analysis

**DOI:** 10.1186/s12886-018-0699-0

**Published:** 2018-02-06

**Authors:** Yana Fu, Qi Dai, Liwei Zhu, Shuangqing Wu

**Affiliations:** 1 The Eye Hospital of Wenzhou Medical University, 270 Xueyuan West Road, Wenzhou City, 325027 Zhejiang Province People’s Republic of China; 2 Department of Ophthalmology, Hangzhou Red Cross Hospital, Hangzhou City, 310003 Zhejiang Province People’s Republic of China

**Keywords:** Antidepressants, Cataract, Meta-analysis, Risk

## Abstract

**Background:**

Epidemiological studies suggest that antidepressants use may increase the risk of cataract, but the results are inconclusive. We aimed to examine this association by performing a systematic review and meta-analysis.

**Methods:**

Relevant studies were identified by searching PubMed and Web of Science databases through June 2017. We included studies that reported risk estimates for the association between antidepressants use and cataract risk. A random-effects model was used to calculate the summary odds ratio (OR) with its 95% confidence interval (CI).

**Results:**

We identified seven studies of antidepressants use and risk of cataract involving 447,672 cases and 1,510,391 controls. Overall, the combined ORs (95% CIs) of cataract for selective serotonin reuptake inhibitors (SSRIs), serotonin noradrenalin reuptake inhibitors (SNRIs), and tricyclic antidepressants (TCAs) were 1.12 (1.06–1.19), 1.13 (1.04–1.24), and 1.19 (1.11–1.28), respectively. A certain degree of heterogeneity was observed across studies (*P* < 0.001, *I*^*2*^ = 92.2% for SSRIs, *P* = 0.026, *I*^*2*^ = 67.5% for SNRIs, and *P* = 0.092, *I*^*2*^ = 58.0% for TCAs).

**Conclusion:**

This meta-analysis provides evidence of a significant positive association between antidepressants use and risk of cataract. Because of the heterogeneity and limited eligible studies, further prospective studies are warranted to confirm the preliminary findings of our study.

## Background

Cataract is defined as partial or complete loss of transparency of the crystalline lens and is considered the primary cause of vision loss worldwide [1]. The high prevalence and incidence of cataract have resulted in a large public health burden. Although the actual mechanism of cataract development remains unclear, several risk factors have been established, including age [2], corticosteroid use [3], hypertension [4], smoking [5], and so on.

Recently, emerging epidemiological studies have focused on the risk of cataract formation of antidepressants. Two population-based studies from Canada [6] and the United States [7] suggested a significant positive association between the use of selective serotonin reuptake inhibitors (SSRIs) and the incidence of cataract. Beaver Dam Eye study showed a tendency toward an increased risk of cataract in users of amitriptyline, a tricyclic antidepressant (TCA) [8]. On the other hand, Becker et al. [9] failed to find a positive relationship between SSRIs and cataract risk using the UK-based Clinical Practice Research Datalink (CPRD).

Given the inconsistency and conflict of the existing literature and the insufficient statistical power of individual studies, we performed the present meta-analysis based on all eligible epidemiological studies that provided data on the association of antidepressants use with cataract risk.

## Methods

### Literature search

We performed this meta-analysis in accordance with the Meta-Analysis of Observational Studies in Epidemiology guidelines [10]. A systematic literature search was carried out in PubMed and Web of Science databases through June 2017 by using the following search strategy: (“antidepressant” or “depression” or “selective serotonin reuptake inhibitor” or “SSRI” or “monoaminoxidase inhibitor” or “MAOI” or “tricyclic antidepressant” or “TCA” or “serotonin noradrenalin reuptake inhibitor” or “SNRI” or “serotonin antagonist and reuptake inhibitor” or “SARI” or “norepinephrine dopamine reuptake inhibitor” or “NDRI” or “norepinephrine reuptake inhibitor” or “NRI” or “noradrenergic and specific serotonergic antidepressant” or “NaSSA”) and “Cataract”, with no restrictions. Cited references of the retrieved articles and reviews were also checked.

### Study selection

Studies included in this meta-analysis met the following criteria: 1) had cohort, nested case-control or case-control study design; 2) the exposure of interest was antidepressants use, including SSRIs, TCAs, serotonin noradrenalin reuptake inhibitors (SNRIs), and so on; 3) the endpoint of interest was cataract incidence; and 4) the risk estimate and the corresponding 95% confidence interval (CI) were reported. If multiple studies used the same population, we included the study with the largest sample size.

### Data extraction and quality assessment

We extracted the following data using a standardized data-collection form: last name of the first author, publication year, study region, number of cases and controls, method of exposure and endpoint assessment, types of antidepressants, risk estimates from the most fully adjusted model and the corresponding 95% CIs, matched or adjusted potential confounders.

We assessed the quality of individual studies using Newcastle-Ottawa Scale (NOS), a 9-star system which consists of three dimensions: selection (four items), comparability (one item), and exposure/outcome (three items) (http://www.ohri.ca/programs/clinical_epidemiology/oxford.asp). Two authors (QD and YF) independently performed the literature search, study selection, data extraction, and quality assessment. Any disagreements were resolved by consensus.

### Statistical analysis

A DerSimonian and Laird random-effects model [11], which considered both within- and between-study variation, was used to calculate the combined estimate of effect size. For studies that separately provided estimated risk estimates for a number of categories of exposure compared with a single reference category, we combined these risk estimates within each study using the method reported by Hamling et al.’s study [12]. Homogeneity across included studies was tested by *Q* statistics at the *P* < 0.10 level of significance [13]. The *I*^*2*^ score, a quantitative measure of inconsistency across studies, was also calculated [13]. Potential publication bias was assessed by a visual funnel plot [14]. All analyses were performed by using STATA version 10.0 (StataCorp, College Station, TX). A *P* value < 0.05 was considered statistically significant, except where otherwise specified.

## Results

### Literature search and study characteristics

**Fig. 1:**
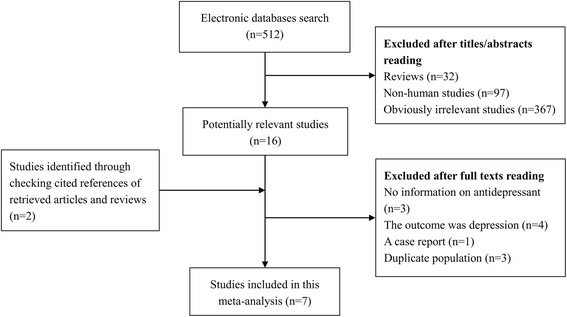
Flow chart of study selection

A flow chart showing the study selection process in detail is presented in Fig. 1. Seven studies [6–9, 15–17] were finally included in this meta-analysis. Of these, five studies were performed in North America, one in Europe, and one in Asia. All individual studies were case-control studies, of which four were nested case-control studies. Results for SSRIs were presented in six of these studies, four for SNRIs, and three for TCAs. These studies were published between 2001 and 2017. Information on exposure and endpoint were mainly collected from medical records. The NOS scores ranged from six to eight, with a mean value of 6.7. The main characteristics of all included studies have been summarized in Table 1.

**Table 1:** Main characteristics of the studies included in this meta-analysis

Author	Year	Region	Design	Drug type	No. of cases	No. of controls	Exposure measurement	Outcome ascertainment	NOS	Matched or adjusted variables
Becker et al.	2017	United Kingdom	Case-control	SSRI, TCA, SNRI, and MAOI	206,931	206,931	Computer records (CPRD)	Computer records (CPRD)	8	Calendar time (same index date), age, sex, general practice, and number of years of active history in the CPRD before the index date, BMI, smoking, diabetes, hypertension, and systemic steroids
Chou et al.	2017	Taiwan	Nested case-control	SSRI, TCA, and SNRI	7651	6637	Computer records (NHIRD)	Computer records (NHIRD)	8	Age, sex, index date, patient’s demographics, mental illness characteristics, propensity score derived from comorbid conditions, and concomitant medications
Erie et al.	2014	United States	Case-control	SSRI	6024	6024	Medical records	Medical records	7	Age, sex, and date of surgery
Wise et al.	2014	United States	Nested case-control	SSRI	45,065	450,650	Medical records (IMS LifeLink database)	Medical records (IMS LifeLink database)	6	Age, time of cohort entry, and follow-up time
Pakzad-Vaezi et al.	2013	Canada	Nested case-control	SSRI	162,501	650,004	Medical records (British Columbia Ministry of Health)	Medical records (British Columbia Ministry of Health)	6	Age, time of cohort entry, and follow-up time
Etminan et al.	2010	Canada	Nested case-control	SSRI and SNRI	18,784	187,840	Medical records	Medical records	6	Age, cohort entry, gender, hypertension, antihypertensive, antidiabetics, statins, and all forms of corticosteroids
Klein et al.	2001	United States	Case-control	TCA	716	2305	Medical history questionnaire	Diagnosed by an ophthalmologist	6	Age and gender

*No.* number, *NOS* Newcastle-Ottawa Scale, *y* year, *SSRI* selective serotonin reuptake inhibitor, *TCA* tricyclic antidepressant, *SNRI* serotonin noradrenalin reuptake inhibitor, *MAOI* monoaminoxidase inhibitor, *NHIRD* National Health Insurance Research Database, *BMI* body mass index, *CPRD* Clinical Practice Research Datalink

### Main analysis by antidepressant classifications

**Fig. 2:**
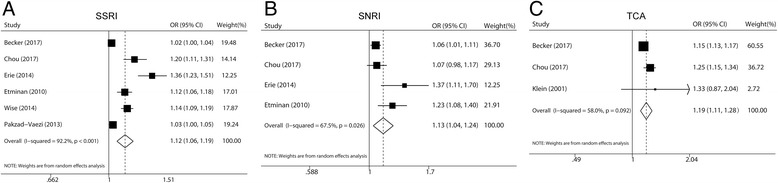
Random-effects meta-analysis of studies that examined three types of antidepressants use may increase the risk of cataract. **a** SSRIs use; **b** SNRIs use; **c** TCAs use

The multivariable-adjusted odds ratios (ORs) for each study and the pooled ORs for the any exposure versus none of antidepressants are presented in Fig. 2. Overall, the combined ORs (95% CIs) of cataract for SSRIs, SNRIs, and TCAs were 1.12 (1.06–1.19), 1.13 (1.04–1.24), and 1.19 (1.11–1.28), respectively. Obvious heterogeneity was found across studies (*P* < 0.001, *I*^*2*^ = 92.2% for SSRIs, *P* = 0.026, *I*^*2*^ = 67.5% for SNRIs, and *P* = 0.092, *I*^*2*^ = 58.0% for TCAs).

### Subgroup analyses by individual antidepressant drugs

**Fig. 3:**
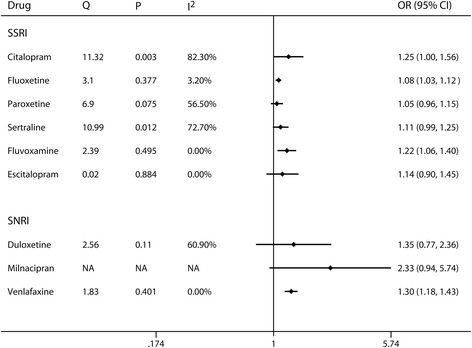
Subgroup analyses by individual antidepressant drugs

The results of subgroup analysis according to individual antidepressant drugs are presented in Fig. 3. For SSRIs antidepressant drugs, a significantly direct association with cataract incidence was observed for fluoxetine (RR 1.08, 95% CI 1.03–1.12) and fluvoxamine (RR 1.22, 95% CI 1.06–1.40). No evidence of association was found for the rest of SSRIs drugs. For SNRIs antidepressant drugs, the combined ORs (95% CIs) of cataract for any exposure versus none were 1.35 (0.77–2.36), 2.33 (0.94–5.74), and 1.30 (1.18–1.43) for duloxetine, milnacipran, and venlafaxine, respectively.

### Publication bias

**Fig. 4:**
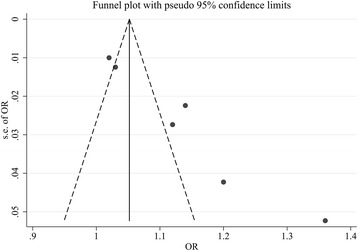
A visual funnel plot to qualitatively assess the publication bias

As only seven studies were included in the present meta-analysis, Begg’s test [18] and Egger’s test [14] were not eligible for publication bias analysis. Hence we adopted a visual funnel plot to qualitatively assess the publication bias. As shown in Fig. 4, a certain degree of asymmetry was observed, which indicated slight publication bias.

## Discussion

This meta-analysis of seven eligible studies involving 447,672 cases and 1,510,391 controls supports a significant positive association of SSRIs, SNRIs and TCAs use with risk of cataract. To the best of our knowledge, this is the first systematic review and meta-analysis aimed to evaluate the relationship between antidepressants use and risk of cataract development.

Heterogeneity is often a concern in a meta-analysis. In the present study, obvious heterogeneity was observed among most analyses, which was partially explained by the following factors: study design was different. Although most included studies were performed in Western countries, population characteristics still varied in genetic and environmental background, antidepressants use, and matched or adjusted confounders.

Several mechanisms may be involved in the positive association of antidepressants use with cataract risk. In animal models, serotonin has been reported to play a crucial role in lens transparency [19]. Elevated serotonin levels have been shown to lead to lens opacity in rats [20]. Similarly, cataract and glaucoma patients also had increased levels of serotonin in the aqueous humor [21]. In addition, serotonin 5-HT1A, 5-HT2A/2C, and 5-HT7 receptors have been identified in the crystalline lens, which participate in regulation of intraocular pressure (IOP) homeostasis [22]. Increased IOP is able to contribute to glaucoma, which is a risk factor for cataract formation [23]. TCAs use is reported to be related with photosensitivity to ultraviolet or sunlight. This latter exposure has been suggested to be associated with cortical cataract in Beaver Dam Eye Study [24]. On the other hand, TCAs is able to inhibit norepinephrine uptake, which may also have cataractogenic properties [25].

Our study had some important strengths. Considering individual studies had limited statistical power, this meta-analysis of seven studies involving a large number of cases and controls improved the power to detect a potential association and provided more robust estimates. Most of the original studies matched or adjusted a series of variables, which greatly reduced the likelihood of confounding bias.

Potential limitations of our study should be considered. First, the number of eligible studies was limited, especially in some subgroup analyses, which might influence the reliability of the results. Second, significant heterogeneity was observed among included studies, which might distort the conclusion of our study. Third, a certain degree of publication bias was observed. Gray literature (e.g., conference abstract) was difficult to find and studies with null results were less likely to be published. Finally, random misclassification of antidepressants might influence the results.

## Conclusion

Use of antidepressants, including SSRIs, SNRIs and TCAs, is associated with an increased risk of cataract development. Considering the huge heterogeneity and limited included studies, further large well-designed prospective studies are warranted to confirm the preliminary findings of our study.

## Abbreviations

95% CI: 95% Confidence Interval
NOS: Newcastle-Ottawa Scale
OR: Odds Ratio
SNRIs: Serotonin noradrenalin reuptake inhibitors
SSRIs: Selective serotonin reuptake inhibitors
TCAs: Tricyclic antidepressants
